# Residual inflammatory risk post STEMI: high prevalence despite LDL-C control and association with other secondary prevention targets

**DOI:** 10.3389/fcvm.2026.1758499

**Published:** 2026-05-29

**Authors:** Ahmed Hassan, Amr Yosry Emam, Mina Samir, Kerolos Sobhy, Ihab Abd El Nabi, Hala Ali, Mohammed Thabet, Ahmed Elghazoly, Nagwa Thabet, Ahmed Elguindy

**Affiliations:** 1 Cairo University Kasr Alainy Faculty of Medicine, Cairo, Egypt; 2 Magdi Yacoub Heart Foundation-Aswan Heart Centre, Cairo, Egypt; 3 Aswan University Faculty of Medicine, New Aswan City, Egypt

**Keywords:** hs-CRP, inflammation, medication adherence, PPCI, residual risk, risk factors, secondary prevention, STEMI

## Abstract

**Background:**

Residual inflammation remains a major contributor to cardiovascular risk after ST-elevation myocardial infarction (STEMI), even with optimal control of traditional risk factors. This study evaluated the prevalence of residual inflammatory risk, using high-sensitivity C-reactive protein (hs-CRP) as a marker, and its association with achieving guideline-recommended secondary prevention targets following primary percutaneous coronary intervention (pPCI).

**Methods:**

We retrospectively analyzed 878 patients who underwent pPCI for STEMI between October 2016 and May 2023. At a mean of 2.4 years post-procedure, hs-CRP and secondary prevention metrics were assessed. Elevated hs-CRP level was defined as greater than 3 mg/L. Multivariable logistic regression was used to determine if there was an association between meeting these clinical targets and having lower hs-CRP levels.

**Results:**

The median hs-CRP level was 2.7 (IQR: 1.2–5), and 44.6% of patients had levels >3 mg/L. Achieving targets for waist circumference, LDL-C (<55 mg/dL), and triglycerides (<150 mg/dL), as well as adherence to statin and ACEI/ARB therapy, were independently associated with lower odds of elevated hs-CRP. Notably, 29.8% of patients with LDL-C < 55 mg/dL still exhibited elevated hs-CRP, indicating persistent inflammatory risk.

**Conclusion:**

Residual inflammatory risk is highly prevalent after STEMI, even among patients with optimal LDL-C control. Comprehensive secondary prevention—including lipid management, central obesity reduction, and medication adherence—correlates with lower hs-CRP. These findings underscore the need for strategies targeting inflammation alongside traditional risk factor control.

## Background

Despite significant advancements in medical and interventional management, cardiovascular disease remains a leading cause of death and disability globally (1). Inflammation plays a crucial role in the development of atherosclerosis, a key factor in coronary artery disease (CAD) and its complications (2). High-sensitivity C-reactive protein (hs-CRP) is a widely used inflammatory marker in clinical practice. It has been identified as an independent predictor of adverse cardiovascular events in patients with CAD (3, 4).

Previous studies have linked hs-CRP levels in patients with ST-Elevation Myocardial Infarction (STEMI) to increased cardiovascular risks and mortality (5, 6). In some earlier studies, hs-CRP has also been associated with an increased risk of recurrent cardiovascular events in stable coronary artery disease (7, 8). However, the association between hs-CRP levels and the status of secondary prevention targets following acute coronary events remains inadequately addressed.

We hypothesize that in patients with a history of primary percutaneous coronary intervention (pPCI), a single-point measurement of hs-CRP will correlate with the concurrent status of secondary prevention targets. This cross-sectional study examines the level of hs-CRP and its relation to the key secondary prevention goals. In addition to representing residual inflammatory risk independent of other factors, we propose that hs-CRP levels may reflect a patient's overall adherence and response to secondary prevention measures, encompassing the complex interplay between traditional risk factors and residual inflammation. This investigation could inform the development of more personalized and effective risk-reduction strategies in this population.

## Methods

### Setting and design

This retrospective analysis of a prospective registry of patients who underwent pPCI at our hospital and subsequently attended follow-up visits. The study aimed to characterize residual inflammatory risk by using hs-CRP as a surrogate marker and its association with the status of secondary prevention targets at a single time point during the follow-up. The local registry included all clinical data and secondary prevention target status for each patient in addition to the measurements of hs-CRP levels. Our ethical committee approved this study protocol.

### Study population

**Figure 1:**
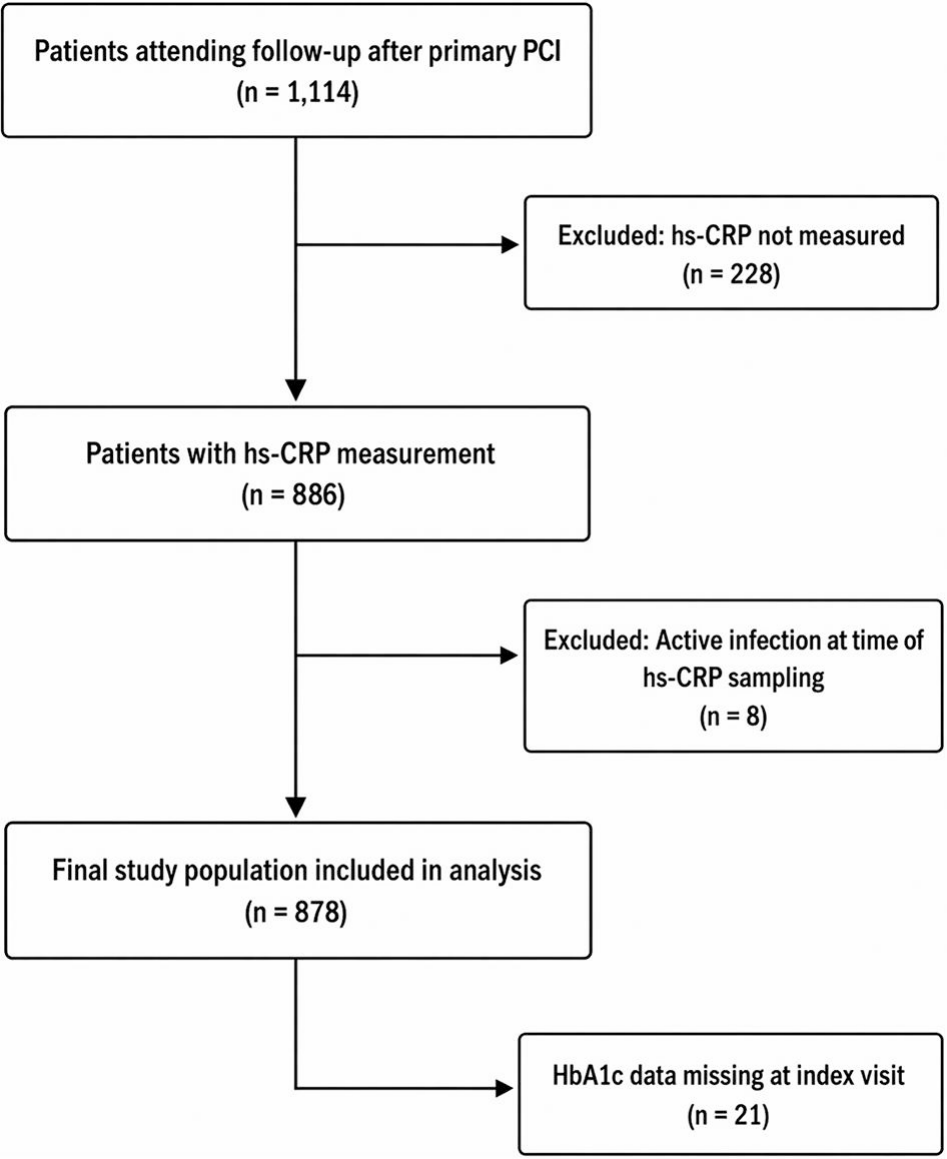
Participant flow diagram. Of 1,114 patients attending follow-up after primary percutaneous coronary intervention, 886 had hs-CRP measured. Eight patients were excluded due to active infection at the time of sampling, resulting in a final study population of 878 patients. HbA1c data were unavailable for 21 patients at the index visit.

The initial cohort included 1,114 patients attending follow-up after primary PCI. Of these, 886 had hs-CRP measured. Eight patients with active infection at the time of sampling were excluded, resulting in a final study population of 878 patients. HbA1c data were missing in 21 patients at the index visit (Figure 1). Given the low proportion of missing data and the observational design, analyses were performed using complete cases without imputation. The visit at which hs-CRP was measured was defined as the index visit, occurring at a mean of 2.4 ± 1.2 years after the initial (index) pPCI. HbA1c data were available for 857 patients, as values were missing for 21 individuals at the index visit.

### Measurements and endpoints

Demographic and clinical data, including age, sex, cardiovascular risk factors, and comorbidities, were collected. Data on STEMI, pPCI characteristics, and routine follow-up visits were extracted from a dedicated electronic database. The achievement of guideline-recommended secondary prevention targets was a key endpoint, assessed at the index follow-up visit. This analysis encompassed the following metrics: complete smoking cessation; a body mass index (BMI) of less than 25 kg/m^2^; waist circumference below sex-specific thresholds (<94 cm for males and <80 cm for females); office blood pressure controlled to under 130/80 mmHg; a low-density lipoprotein cholesterol (LDL-C) level of less than 55 mg/dL; and for diabetic patients, glycemic control defined by a glycated hemoglobin (HbA1c) value of ≤7%.

### Laboratory measurement of hs-CRP

Serum hs-CRP concentration was measured using the reagent CRPHS (Roche, Switzerland) and the autoanalyzer Cobas C311 (Roche, La Roche Ltd, Switzerland). Other markers were determined using routine laboratory methods. For categorization in our study, Elevated hs-CRP was defined as level > 3 mg/L. This cutoff was selected based on CDC and AHA consensus recommendations and on its adoption in cardiovascular risk-stratification studies to define high inflammatory risk (9).

### Statistical analysis

Descriptive statistics were utilized to summarize the characteristics of the patients. Continuous variables are presented as the mean ± standard deviation (SD), while categorical variables are shown as frequencies and percentages. To evaluate the differences in baseline characteristics between patients with elevated hs-CRP levels (>3 mg/L) and those with low hs-CRP levels (≤3 mg/L), the chi-square test was employed for categorical variables, and either the student's *t*-test or the Mann–Whitney *U-*test (if normality assumptions were not met) was used for continuous variables. Additionally, to identify independent predictors of elevated hs-CRP levels (>3 mg/L), a binary logistic regression analysis was conducted, with hs-CRP status as the dependent variable. A separate multivariable logistic regression analysis was performed for the subgroup of patients with LDL-C < 55 mg/dL to identify predictors of elevated hs-CRP within this group. The same variables were included in the model, with the addition of HbA1c (<7% vs. ≥7%) and adjusted for age and sex.

## Results

### Patients characteristics

The study cohort consisted of 878 patients who underwent pPCI at our hospital between October 2016 and May 2023. The mean age was 55 ± 11 years, with males comprising 734 (83.6%). The mean duration from the index pPCI to the index visit was 2.4 ± 1.2 years. The mean hs-CRP level in the entire cohort was 3.4 ± 2.6 mg/L. Baseline patient characteristics and their association with hs-CRP levels at follow-up are presented in Table 1. Female sex, hypertension, and diabetes were each associated with significantly higher hs-CRP levels.

**Table 1:** Baseline characteristics and secondary prevention target achievement, stratified by hs-CRP level.

Variables	Overall (*N* = 878)	hsCRP > 3 mg/L (*N* = 392)	hsCRP ≤ 3 mg/L (*N* = 486)	*p*-value
Baseline Characteristics				
Age, mean ± SD	55 ± 11 years	56 ± 11	55 ± 11	0.084
Gender: Male, *n* (%)	734 (83.6%)	273 (69.6%)	461 (94.8%)	**<0**.**001**
Hypertension: yes, *n* (%)	565 (64.3%)	270 (68.9%)	295 (60.7%)	**0**.**006**
Diabetes: yes, *n* (%)	371 (42.3%)	177 (45.2%)	194 (40.0%)	0.066
Current Smoker at Index STEMI: yes, *n* (%)	589 (67.1%)	271 (69.1%)	318 (65.5%)	0.355
Secondary Prevention Targets (at Index Visit)				
Controlled BP (<130/80 mmHg), *n* (%)	312 (35.5%)	129 (32.9%)	183 (37.7%)	0.156
HbA1c < 7%, *n* (%)^a^ (*N* = 857)	520 (60.8%)	218 (55.6%)	302 (62.1%)	0.147
LDL < 55 mg/dL, *n* (%)^a^(*N* = 869)	141 (16.2%)	42 (10.7%)	99 (20.4%)	**<0**.**001**
Triglycerides <150 mg/dL, *n* (%)	462 (52.6%)	183 (46.7%)	279 (57.4%)	**0**.**002**
Smoking Cessation: yes, *n* (%)	254 (28.9%)	109 (27.8%)	145 (29.9%)	0.612
BMI < 25 kg/m^2^, *n* (%)	222 (25.3%)	83 (21.2%)	139 (28.6%)	**0**.**004**
Waist Circumference (within target), *n* (%)	171 (19.5%)	54 (13.8%)	117 (24.1%)	**<0**.**001**
Medications (at Index Visit)				
Statin Use: yes, *n* (%)	617 (70.3%)	252 (64.3%)	365 (75.1%)	**0**.**001**
ACEI/ARB Use: yes, *n* (%)	514 (58.5%)	205 (52.3%)	309 (63.6%)	**0**.**003**
Aspirin Use: yes, *n* (%)	824 (93.8%)	371 (94.6%)	453 (93.2%)	0.400
P2Y12 Inhibitor Use: yes, *n* (%)	164 (18.7%)	74 (18.9%)	90 (18.5%)	0.936

A *p*-value less than 0.05 is considered statistically significant.

a Sample sizes differ slightly for these variables as they were analyzed separately. A *p*-value less than 0.05 is generally considered statistically significant.

Bold values indicate statistically significant results (*p* < 0.05).

### Association of elevated hs-CRP level with risk factor control and medication adherence

The associations between categorized hs-CRP levels (>3 mg/L vs. ≤3 mg/L) and secondary prevention targets are detailed in Table 3. Elevated hs-CRP levels were significantly associated with uncontrolled blood pressure, elevated BMI, and LDL-C ≥ 55 mg/dL (Table 1).

As shown in Table 2, hs-CRP levels differed significantly across several baseline characteristics, secondary prevention targets, and medication categories. Women had higher hs-CRP levels than men (4.4 ± 2.8 vs. 3.2 ± 2.6 mg/L, *p* < 0.001). Higher hs-CRP levels were also observed in patients with hypertension [median 3.01 [IQR 1.5–5.3] vs. 2.45 [1.1–4.7] mg/L, *p* = 0.006] and diabetes [2.91 [1.5–5.7] vs. 2.5 [1.2–4.8] mg/L, *p* = 0.041]. Increased adiposity was associated with higher hs-CRP, including higher levels in patients with BMI ≥ 25 kg/m^2^ compared with BMI < 25 kg/m^2^ [2.9 [1.3–5.2] vs. 2.3 [1.0–3.9] mg/L, *p* < 0.001], and in those with increased waist circumference compared with those within target [3.6 [1.4–5.1] vs. 2.7 [0.9–3.5] mg/L, *p* < 0.001]. Patients who achieved guideline-recommended lipid targets had significantly lower hs-CRP levels, including LDL-C < 55 mg/dL [1.8 [0.9–3.9] vs. 2.9 [1.3–5.0] mg/L, *p* = 0.002] and triglycerides <150 mg/dL [2.4 [1.0–4.5] vs. 3.1 [1.6–5.6] mg/L, *p* < 0.001].

**Table 2:** Distribution of hs-CRP levels across clinical characteristics and secondary prevention measures.

Variables	hsCRP level	*P* value
	mean ± SD or median (IQR)	
Baseline patient characteristics during the index primary PCI:		
Gender		
Male (*n* = 734)	3.2 (± 2.6)	**< 0.001**
Female (*n* = 144)	4.4 (± 2.8)	
Hypertension		
Yes (*n* = 565)	3.01 (1.5–5.3)	**0**.**006**
No (*n* = 313)	2.45 (1.1–4.7)	
Diabetes (*n* = 507)		
Yes (*n* = 371)	2.91 (1.5–517)	**0**.**041**
No (*n* = 518)	2.5 (1.2–4.8)	
Smoking status at index STEMI		
Smoker (*n* = 589)	3.41 (± 2.7)	0.877
Non-Smoker (*n* = 289)	3.44 (± 2.7)	
Secondary prevention targets		
Blood pressure control (<130/80)		
Controlled BP (*n* = 313)	2.4 (1.1–4.8)	0.056
Uncontrolled BP (*n* = 565)	2.8 (1.2–5)	
HbA1c		
<7 (*n* = 340)	2.5 (1.2–5)	0.121
≥7 (*n* = 518)	2.9 (1–4.1)	
Cessation of smoking		
Yes (*n* = 289)	3.34 (± 2.70)	0.459
No (*n* = 588)	3.50 (± 2.71)	
LDL-C level (*n* = 779)		
LDL-C ≥ 55 mg/dL (*n* = 719)	2.9 (1.3–5)	**0**.**002**
LDL-C < 55 mg/dL (*n* = 141)	1.8 (0.9–3.9)	
Serum triglycerides		
≥150 mg/dL	3.1 (1.6–5.6)	**< 0.001**
<150 mg/dL	2.4 (1–4.5)	
Body mass index (BMI)		
BMI ≥ 25 (*n* = 656)	2.9 (1.3–5.2)	**< 0.001**
BMI < 25 (*n* = 222)	2.3 (1–3.9)	
Waist Circumference (Male < 94 cm or Female < 80 cm)		
Yes (*n* = 171)	2.7 (0.9–3.5)	**< 0.001**
No (*n* = 707)	3.6 (1.4–5.1)	
Medical Treatment		
Continuation of Statins therapy		
Yes (*n* = 617)	3.4 (1.1–4.7)	**< 0.001**
No (*n* = 261)	2.4 (1.7–6.2)	
Clopidogrel at time of follow up		
Yes (*n* = 164)	2.8 (1.4–4.9)	0.924
No (*n* = 714)	2.7± (1.2–5)	
Aspirin at time of follow up		
Yes (*n* = 824)	2.8 (1.2–5)	0.594
No (*n* = 54)	2.3 (1.1–5)	
ACEI or ARBs at time of follow up		
Yes (*n* = 514)	2.4(± 1.1–4.8)	**0**.**001**
No (*n* = 364)	3.1± (1.4–5.6)	

Bold values indicate statistically significant results (*p* < 0.05).

**Figure 2:**
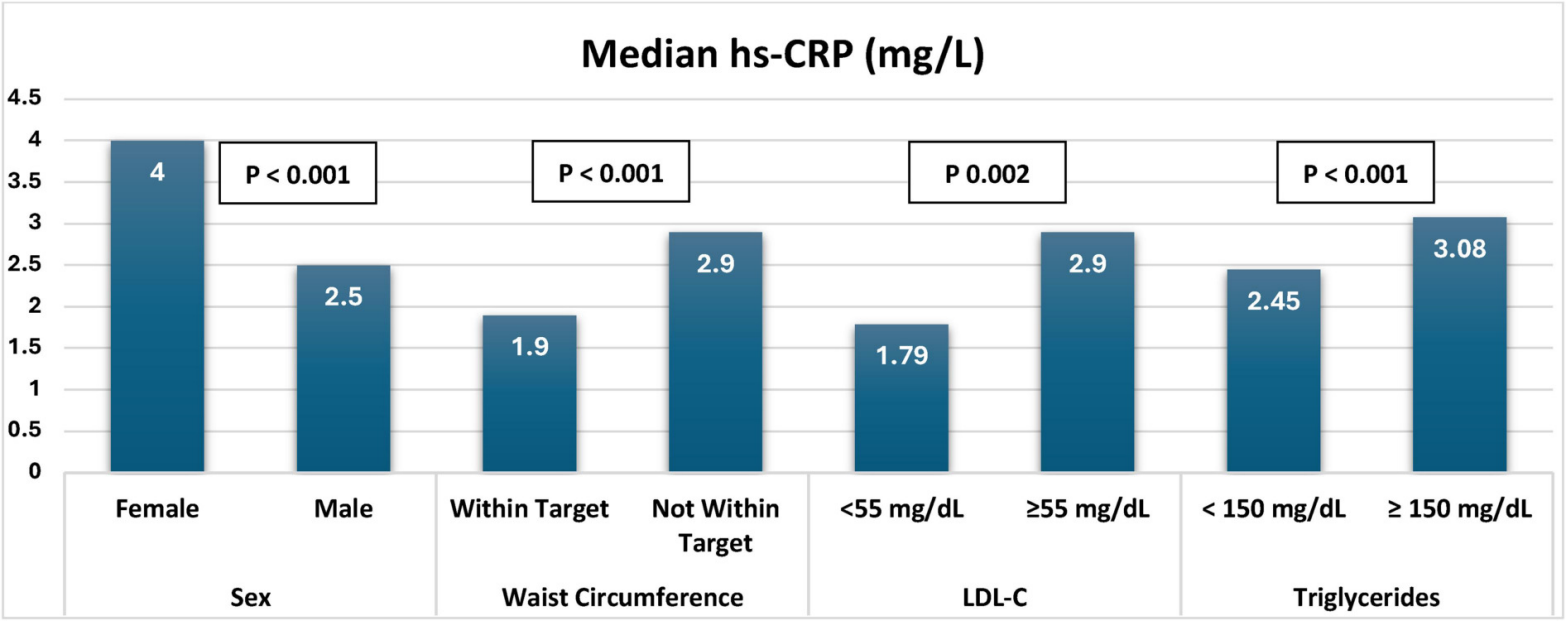
The graph displays the median hs-CRP levels (mg/L) for different categories of secondary prevention targets, including sex, waist circumference, LDL-C, and triglycerides.

We analyzed multivariate binary logistic regression to identify independent predictors of elevated hs-CRP levels (>3 mg/L). The model included the following variables: BMI (<25 kg/m^2^), waist circumference (<94 cm in males or <80 cm in females), LDL-C (<55 mg/dL), triglycerides (< 150 mg/dL), statin use, and ACEI/ARB use and it was adjusted to gender. The results of the multivariate analysis are presented in Table 3. Female sex was significantly associated with higher odds of having elevated hs-CRP (OR = 2.49, 95% CI: 1.69–3.67, *p* < 0.001). Having target levels for waist circumference, LDL-C, and triglycerides were all significantly associated with lower odds of elevated hs-CRP. BMI was not independently associated with elevated hs-CRP. Statin use and ACEI/ARB use (OR = 0.71, 95% CI: 0.51–0.97, *p* = 0.033) were associated with lower odds of elevated hs-CRP. Figure 2 illustrates the median hs-CRP levels across different categories of secondary prevention targets.

**Table 3:** Multivariable logistic regression analysis of factors associated with elevated hs-CRP (>3 mg/L):.

**Variable**	**Odds Ratio (95% CI)**	***p*-value**
Female Sex	2.49 (1.69–3.67)	**<0**.**001**
Secondary prevention target		
BMI ≥ 25	1.10 (0.74–1.64)	0.620
Increased waist Circumference	1.85 (1.21–2.86)	**0**.**005**
LDL > 55 mg/dL	1.89 (1.26–2.83)	**0**.**003**
Triglycerides ≥150 mg/dL	1.52 (1.16–2.00)	**0**.**005**
Medications		
Statin discontinuation	1.45 (1.03–2.04)	**0**.**024**
No ACEI/ARB therapy	1.41 (1.03–1.93)	**0**.**033**

A *p*-value less than 0.05 is considered statistically significant.

Bold values indicate statistically significant results (*p* < 0.05).

### Residual inflammatory risk in patients with optimal LDL-C control

Of the total cohort, 141 patients achieved an LDL-C level of less than 55 mg/dL at follow-up. This subgroup had a median hs-CRP level of 1.8 mg/L. Notably, 42 (29.8%) of these patients with well-controlled LDL-C still exhibited significantly elevated hs-CRP levels (>3 mg/L).

Univariate analysis within this subgroup revealed that hs-CRP levels were significantly higher in female patients, patients with the diagnosis of type 2 diabetes, and patients with HbA1c > 7 mg/dL at follow-up (Table 4). The level of HbA1c > 7 mg/dL in a logistic regression analysis adjusted for gender remained significantly associated with elevated hS-CRP (OR 2.59, 95% CI 1.20–5.59, *p* = 0.015).

**Table 4:** Univariate analysis of factors associated with elevated hs-CRP (>3 mg/L) in patients with optimal LDL-C control.

Variable	Elevated hsCRP	Low hsCRP	*p*-value
	(*n* = 42)	(*n* = 99)	
Female, (*n*, %)	11 (26.2%)	9 (9.1%)	**0**.**008**
Diabetes Mellitus, (*n*, %)	25 (59.5%)	36 (36.4%)	**0**.**011**
Hypertension, (*n*, %)	20 (47.6%)	42 (42.4%)	0.570
Current Smoker, (*n*, %)	26 (61.9%)	16 (16.2%)	0.250
Secondary prevention targets			
BMI ≥ 25, (*n*, %)	29 (69.0%)	73 (73.7%)	0.569
Increased waist Circumference, (*n*, %)	38 (90.5%)	84 (84.8%)	0.432
Triglycerides ≥150 mg/dL, (*n*, %)	24 (57.1%)	59 (59.6%)	0.787
Uncontrolled blood pressure, (*n*, %)	22 (52.4%)	45 (45.4%)	0.451
HbA1c ≥ 7%, (n, %)	22 (52.4%)	29 (29.3%)	**0**.**023**

A *p*-value less than 0.05 is considered statistically significant.

Bold values indicate statistically significant results (*p* < 0.05).

## Discussion

This study demonstrates a high prevalence of residual inflammatory risk, as evidenced by elevated hs-CRP (>3 mg/L), in 44.6% of patients at a mean of 2.4 years post-STEMI. Our analysis identified that achieving targets for waist circumference, LDL-C, and triglycerides, along with adherence to statin and ACEI/ARB therapy, were independently associated with lower odds of elevated hs-CRP. A key finding is that this risk persists even with optimal lipid control, as 29.8% of patients with an LDL-C < 55 mg/dL had elevated hs-CRP.

Inflammation is recognized as a critical driver of residual cardiovascular risk, playing a pivotal role in atherosclerosis pathogenesis (7, 8, 10, 11). Elevated hs-CRP is linked to recurrent cardiovascular events and mortality in stable CAD and post-acute coronary syndrome (12–16). For example, Toso and colleagues reported that among acute coronary syndrome survivors on high-intensity statins, hs-CRP was one of only two biomarkers independently predicting 3-year mortality (17). Also, Mani et al. showed that elevations in hs-CRP levels, both initially and in subsequent measurements over a 16-week period, were correlated with an increased risk of major adverse cardiac events (MACE) (17).

At a mean of 2.4 years post-pPCI, the mean hs-CRP level in our cohort was 3.4 ± 2.6 mg/L. Using a threshold of >3 mg/L to define high residual inflammatory risk—a cutoff that aligns with established risk stratification criteria and facilitates comparison with other studies in similar populations ^(^9). CRP levels vary by sex and race, with higher values observed in African Americans than in Caucasians and in females compared with males (18). In young adults, the median baseline CRP level is 0.8 mg/L; the 90th percentile is 3.0 mg/L, and the 99th percentile is 10 mg/L (19). While we defined residual inflammatory risk using an hs-CRP threshold of >3 mg/L to identify patients at clearly heightened cardiovascular risk, emerging evidence supports the clinical relevance of lower hs-CRP thresholds in statin-treated ASCVD populations. In trials such as CANTOS (20) and COLCOT (21), hs-CRP levels ≥2 mg/L identified patients who derived benefit from targeted anti-inflammatory therapy despite optimal lipid control. Thus, hs-CRP > 2 mg/L may represent an intermediate-risk inflammatory phenotype, whereas hs-CRP > 3 mg/L denotes a more pronounced residual inflammatory burden. Although continuous modeling may offer statistical advantages, dichotomous classification retains clinical relevance by identifying patients with high inflammatory burden who may benefit from intensified preventive strategies.

Although women comprised a smaller proportion of the cohort, reflecting the epidemiology of STEMI in younger populations in the region, female sex was independently associated with higher hs-CRP levels. Prior studies (22–26) have demonstrated sex-related differences in inflammatory profiles, potentially driven by differences in adiposity distribution, hormonal factors, and metabolic risk. While some studies report higher hs-CRP in women ^(^27, 28), the magnitude of the association between hs-CRP and coronary heart disease risk may be less pronounced in women than in men, as shown in a meta-analysis by the Emerging Risk Factors Collaboration (29). These findings should be interpreted cautiously and warrant validation in cohorts with greater female representation. Consistent with previous studies (22–26), hs-CRP levels were higher in those with hypertension or diabetes at baseline.

Central adiposity emerged as a key driver of inflammation in our analysis. While both BMI and waist circumference were associated with lower hs-CRP in univariate analysis, only waist circumference remained a significant predictor in the multivariate model. This finding aligns with evidence that visceral fat is metabolically active and secretes pro-inflammatory cytokines (e.g., IL-6) that raise systemic CRP levels (27, 30–34).

In terms of lipid management, having LDL-C of less than 55 mg/dL and adherence to statin therapy were independently associated with lower hs-CRP. This aligns with the known pleiotropic anti-inflammatory effects of statins (35), as demonstrated in trials like JUPITER trial (36), in which rosuvastatin reduced LDL-C levels by 50% and hs-CRP levels by 37% in patients with normal to low cholesterol but high hs-CRP levels. Notably, the anti-inflammatory effect of lipid-lowering therapy may be partly independent of the magnitude of LDL-C reduction (37). Similarly, achieving target triglyceride levels was associated with lower hs-CRP, supporting the link between hypertriglyceridemia and inflammation (38, 39). The mechanisms by which triglycerides promote inflammation are complex, including direct stimulation of pro-inflammatory cytokine production, impairment of endothelial function, and activation of intracellular inflammatory signalling (40).

Furthermore, ACE inhibitors/ARBs were also independently associated with lower hs-CRP after adjusting for age and secondary prevention targets, consistent with evidence of anti-inflammatory effects beyond blood pressure control, possibly via modulation of angiotensin II pathways (41). While not fully elucidated, these anti-inflammatory mechanisms may also involve oxidative stress and endothelial function modulation. While achieving blood pressure targets and smoking cessation showed univariate associations with lower hs-CRP, these were not significant in the multivariate analysis. This may be due to limited statistical power or residual confounding.

A particularly compelling finding was the persistence of elevated hs-CRP in patients achieving optimal LDL-C control. Nearly one-third of patients with LDL-C < 55 mg/dL had hs-CRP levels >3 mg/L, highlighting a dissociation between lipid-mediated and inflammation-mediated risk pathways. The elevated hs-CRP in patients with well-controlled LDL-C could be attributed to several factors, including persistent inflammation from other uncontrolled risk factors, inadequate control of different lipid parameters (e.g., non-HDL cholesterol), and genetic predisposition. The CANTOS trial validates this pathway, demonstrating that directly targeting interleukin-1β with canakinumab reduced cardiovascular events independent of LDL-C (20). This supports the concept that inflammation independent of LDL-C contributes significantly to residual cardiovascular risk.

Recent studies have further refined the concept of residual inflammatory risk beyond traditional lipid-centric paradigms (42, 43). Contemporary analyses demonstrate that persistent hs-CRP elevation identifies a subgroup of post-ACS patients at heightened risk despite optimal LDL-C control, and emerging evidence links elevated inflammatory biomarkers to adverse presentations such as cardiogenic shock and hemodynamic instability (44).

Our findings extend this evolving body of literature by demonstrating, in a real-world post-STEMI cohort, that residual inflammation remains prevalent years after the index event and clusters with modifiable metabolic and therapeutic factors. Our findings have several clinical implications. First, hs-CRP measurement can identify patients with persistent inflammatory risk who may benefit from intensified monitoring and aggressive management of all modifiable factors, including central obesity, hypertriglyceridemia, and hyperglycemia. Second, in patients on optimal statin therapy, hs-CRP may provide prognostic information beyond LDL-C (20, 45). Finally, our data support guideline considerations for enhanced risk stratification using hs-CRP in chronic coronary syndromes and the potential role of targeted anti-inflammatory therapies, such as low-dose colchicine (46).

## Conclusion

Residual inflammatory risk, defined by hs-CRP > 3 mg/L, remains highly prevalent in real-world post-STEMI patients, even among those achieving guideline-recommended LDL-C targets and receiving optimal medical therapy. Achievement of guideline-recommended secondary prevention goals—including central obesity reduction, triglyceride control, and adherence to statin and ACEI/ARB therapy—is associated with lower inflammatory burden. These findings highlight the importance of a comprehensive, multimodal approach to secondary prevention that addresses inflammatory risk alongside traditional cardiovascular targets.

## Limitations

This study has several limitations. Its retrospective, cross-sectional design precludes causal inference and is subject to residual confounding. The single-center setting may limit generalizability. Hs-CRP was assessed at a single follow-up time point, and intra-individual variability cannot be excluded. The absence of baseline hs-CRP and longitudinal outcome data limits assessment of temporal dynamics and prognostic implications. Although missing data were minimal, complete-case analysis may introduce bias. Finally, while hs-CRP > 3 mg/L was used to define high residual inflammatory risk, future studies should explore alternative inflammatory and lipid thresholds to further refine risk stratification.

## Data Availability

The raw data supporting the conclusions of this article will be made available by the authors upon reasonable request.
